# Supplementary material for: Exploring the Role of Guanylate-Binding Protein-2 in Activated Microglia-Mediated Neuroinflammation and Neuronal Damage
Source: Biomedicines. 2024 May 20;12(5):1130. doi: 10.3390/biomedicines12051130 (PMC11117630; doi:10.3390/biomedicines12051130)
Supplement: Supplementary file 1 [file biomedicines-12-01130-s001.zip › biomedicines-2996288-supplementary.pptx]

## Slide 1
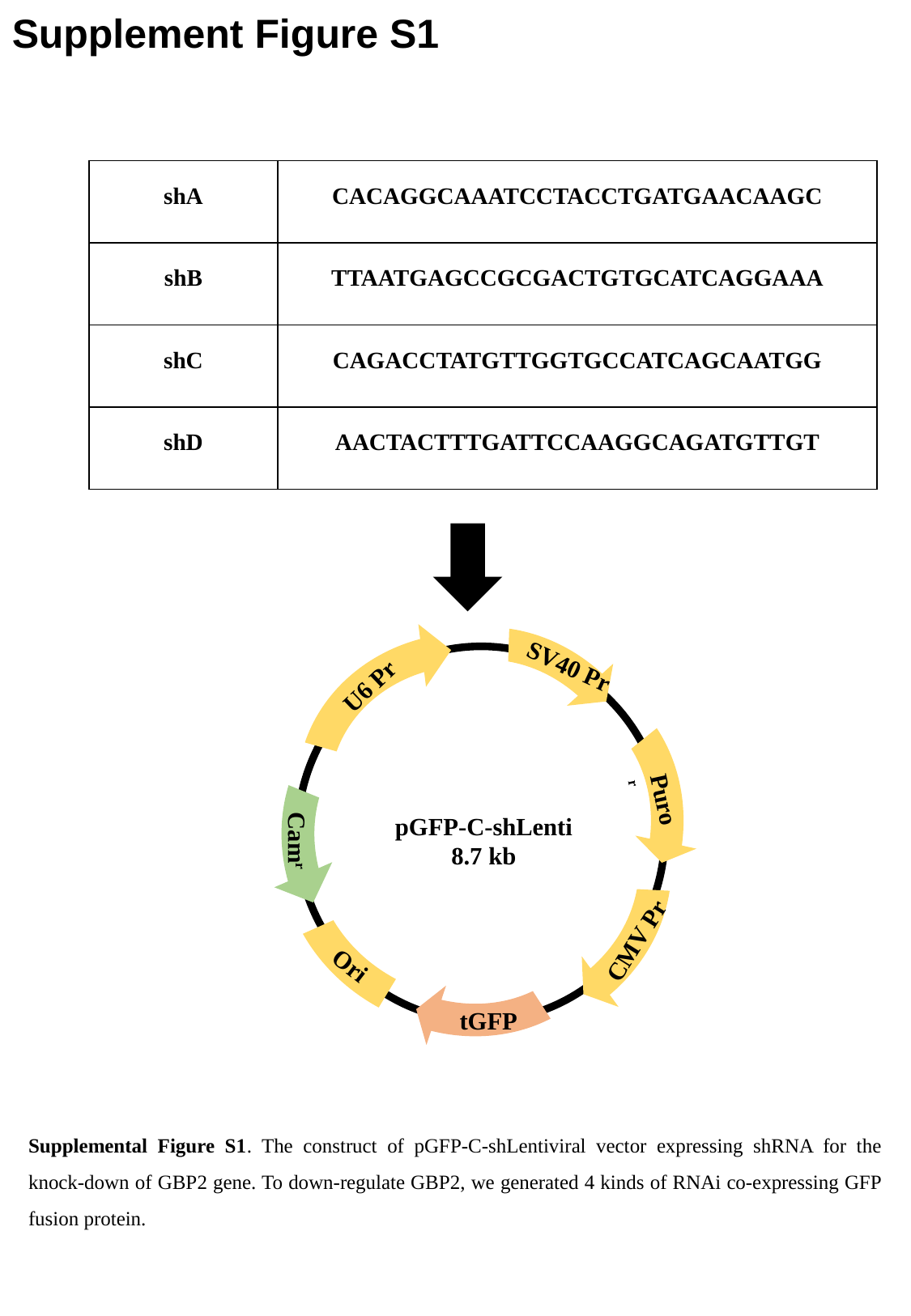

Supplement Figure S1
| shA | CACAGGCAAATCCTACCTGATGAACAAGC |
| --- | --- |
| shB | TTAATGAGCCGCGACTGTGCATCAGGAAA |
| shC | CAGACCTATGTTGGTGCCATCAGCAATGG |
| shD | AACTACTTTGATTCCAAGGCAGATGTTGT |
SV40 Pr
U6 Pr
Puror
pGFP-C-shLenti
8.7 kb
Camr
CMV Pr
Ori
tGFP
Supplemental Figure S1. The construct of pGFP-C-shLentiviral vector expressing shRNA for the knock-down of GBP2 gene. To down-regulate GBP2, we generated 4 kinds of RNAi co-expressing GFP fusion protein.
